# Supplementary material for: Chromatix: a differentiable, GPU-accelerated wave-optics library
Source: Nat Methods. 2026 Jun 8;23(7):1388–98. doi: 10.1038/s41592-026-03121-x (PMC13345901; doi:10.1038/s41592-026-03121-x)
Supplement: Supplementary file 2 — Peer Review File [file 41592_2026_3121_MOESM2_ESM.pdf]

nature portfolio

Peer Review File

Chromatix: a differentiable, GPU-accelerated wave-optics library

Corresponding Author: Dr Srinivas Turaga

This file contains all editorial decision letters in order by version, followed by all author rebuttals in order by version.

Version 0:

Decision Letter:

18th Sep 2025

Dear Srini,

Thank you for your patience. Your Article, "Chromatix: a differentiable, GPU-accelerated wave-optics library", has now been seen by two reviewers. As you will see from their comments below, although the reviewers find your work of considerable potential interest, they have raised a number of concerns. We continue to be interested in potentially publishing your manuscript in Nature Methods, but would like to evaluate your response to these concerns before we reach a final decision on publication. We therefore invite you to revise your manuscript to address these concerns.

We are committed to providing a fair and constructive peer-review process. Do not hesitate to contact us if there are specific requests from the reviewers that you believe are technically impossible or unlikely to yield a meaningful outcome.

When revising your paper:

* include a point-by-point response to the reviewers and to any editorial suggestions

* please underline/highlight any additions to the text or areas with other significant changes to facilitate review of the revised manuscript

* address the points listed described below to conform to our open science requirements

* ensure it complies with our general format requirements as set out in our guide to authors at www.nature.com/naturemethods

* resubmit all the necessary files by using the link below to access your home page

Link Redacted

Note: This URL links to your confidential account and associated information about manuscripts you may have submitted, or that you are reviewing for us. If you wish to forward this email to co-authors, please delete this link.

We hope to receive your revised paper within 1-2 months. If you are substantially delayed, please let us know. In this event, we will still be happy to reconsider your paper at a later date so long as nothing similar has been accepted for publication at Nature Methods or published elsewhere.

OPEN SCIENCE REQUIREMENTS

REPORTING SUMMARY
When revising your manuscript, please update your reporting summary.

Reporting summary: https://www.nature.com/documents/nr-reporting-summary.zip

If your paper includes custom software, we also ask you to complete a supplemental reporting summary.

Software supplement: https://www.nature.com/documents/nr-software-policy.pdf

Please submit these with your revised manuscript. They will be available to reviewers to aid in their evaluation if the paper is re-reviewed. If you have any questions about the checklist, please see http://www.nature.com/authors/policies/availability.html or contact me.

Please note that these forms are dynamic 'smart pdfs' and must therefore be downloaded and completed in Adobe Reader. We will then flatten them for ease of use by the reviewers. If you would like to reference the guidance text as you complete the template, please access these flattened versions at http://www.nature.com/authors/policies/availability.html.

For any revision that includes light microscopy data, we ask our authors to please include a completed light microscopy reporting table [https://www.nature.com/documents/Light_microscopy_reporting_table.xlsx] to ensure the methods are described thoroughly. The table will be available to reviewers and ultimately published should the manuscript be accepted at the journal.

Finally, please ensure that you retain unprocessed data and metadata files after publication, ideally archiving data in perpetuity, as these may be requested during the peer review and production process or after publication if any issues arise.

EXTENDED DATA FIGURES
When re-submitting your manuscript, please ensure that any supplementary figures and tables that are crucial to the manuscript's conclusions are converted into Extended Data figures and tables to increase visibility of these data. Extended Data figures and tables are online-only (present in the online PDF and full-text HTML versions of the paper), peer-reviewed display items that provide essential background to the article but are not included in the main article due to space constraints. A maximum of ten Extended Data display items (figures and tables) is permitted.

DATA AVAILABILITY
We strongly encourage you to deposit all new data associated with the paper in a persistent repository where they can be freely and enduringly accessed. We recommend submitting the data to discipline-specific and community-recognized repositories; a list of repositories is provided here: http://www.nature.com/sdata/policies/repositories

All novel DNA and RNA sequencing data, protein sequences, genetic polymorphisms, linked genotype and phenotype data, gene expression data, macromolecular structures, and proteomics data must be deposited in a publicly accessible database, and accession codes and associated hyperlinks must be provided in the "Data Availability" section.

For papers containing bioimaging data, we strongly recommend depositing the data to Bioimage Archive (https://www.ebi.ac.uk/bioimage-archive/). Associated accession codes and hyperlinks should be provided in the "Data Availability" section.

Refer to our data policies here: https://www.nature.com/nature-research/editorial-policies/reporting-standards#availability-of-data

To further increase transparency, we encourage you to provide, in tabular form, the data underlying the graphical representations used in your figures. This is in addition to our data-deposition policy for specific types of experiments and large datasets. For readers, the source data will be made accessible directly from the figure legend. Spreadsheets can be submitted in .xls, .xlsx or .csv formats. Only one (1) file per figure is permitted: thus if there is a multi-paneled figure the source data for each panel should be clearly labeled in the csv/Excel file; alternately the data for a figure can be included in multiple, clearly labeled sheets in an Excel file. File sizes of up to 30 MB are permitted. When submitting source data files with your manuscript please select the Source Data file type and use the Title field in the File Description tab to indicate which figure the source data pertains to.

Please include a "Data availability" subsection in the Online Methods. This section should inform readers about the availability of the data used to support the conclusions of your study, including accession codes to public repositories, references to source data that may be published alongside the paper, unique identifiers such as URLs to data repository entries, or data set DOIs, and any other statement about data availability. At a minimum, you should include the following statement: "The data that support the findings of this study are available from the corresponding author upon request", describing which data is available upon request and mentioning any restrictions on availability. If DOIs are provided, please include these in the Reference list (authors, title, publisher (repository name), identifier, year). For more guidance on how to write this section please see: http://www.nature.com/authors/policies/data/data-availability-statements-data-citations.pdf

CODE AVAILABILITY
Please include a “Code Availability” subsection in the Online Methods which details how your custom code is made available. Only in rare cases (where code is not central to the main conclusions of the paper) is the statement “available upon request” allowed (and reasons should be specified).

We request that you deposit code in a DOI-minting repository such as Zenodo, Gigantum or Code Ocean and cite the DOI in the Reference list. We also request that you use code versioning and provide a license.

For more information on our code sharing policy and requirements, please see:
https://www.nature.com/nature-research/editorial-policies/reporting-standards#availability-of-computer-code

MATERIALS AVAILABILITY
As a condition of publication in Nature Methods, authors are required to make unique materials promptly available to others without undue qualifications.

Authors reporting new chemical compounds must provide chemical structure, synthesis and characterization details. Authors reporting mutant strains and cell lines are strongly encouraged to use established public repositories.

More details about our materials availability policy can be found at https://www.nature.com/nature-portfolio/editorial-policies/reporting-standards#availability-of-materials

ORCID
Nature Methods is committed to improving transparency in authorship. As part of our efforts in this direction, we are now requesting that all authors identified as ‘corresponding author’ on published papers create and link their Open Researcher and Contributor Identifier (ORCID) with their account on the Manuscript Tracking System (MTS), prior to acceptance. This applies to primary research papers only. ORCID helps the scientific community achieve unambiguous attribution of all scholarly contributions. You can create and link your ORCID from the home page of the MTS by clicking on ‘Modify my Springer Nature account’. For more information please visit please visit www.springernature.com/orcid.

Please do not hesitate to contact me if you have any questions or would like to discuss these revisions further. We look forward to seeing your revised manuscript and thank you for the opportunity to consider your work.

Best regards,
Nina

Nina Vogt, PhD
Senior Editor
Nature Methods

Reviewers' Comments:

Reviewer #1 (Remarks to the Author):

This manuscript introduces Chromatix, an open-source, JAX-based, GPU-accelerated, and differentiable wave-optics simulation library. While many differentiable wave-optics simulators exist, Chromatix generally offers a more complete feature set and, largely (entirely?) due to its JAX-based implementation, is more efficient and parallelizable than existing methods. Chromatix is benchmarked on a range of computational microscopy problems and is shown to offer consistent acceleration of >2x on one GPU and up to 22x on many GPUs. Its parallelism also allows it to tackle larger, higher-resolution problems than existing methods can handle. Chromatix is validated on an impressive number of computational microscopy problems including spatially-varying deconvolution, phase retrieval, PSF engineering, and computer generated holography.

While not groundbreaking (similar though less performant software has existed for some time), Chromatix could still be of value to the computational imaging community. The support for multi-GPU acceleration is a particularly valuable contribution; it enables higher-resolution reconstructions than are possible with alternative methods.

Comments:
The discussion of related work is lacking. It overlooks several recent works that offer capabilities that are not supported by Chromatix. For instance, [A] supports metaoptics design and [B] supports differentiable hybrid ray-wave based methods are fast enough to enable the design of compound optics. The manuscript also overlooks [C] and a large body of follow-up

works that used differentiable end-to-end optical models to design computational imaging systems. Other early works that used differentiable optical models for computational microscopy problems are also overlooked [D]. The discussion of CGH is also somewhat sparse.

[A] D-flat: Hazineh, Dean S., et al. "D-flat: A differentiable flat-optics framework for end-to-end metasurface visual sensor design." arXiv preprint arXiv:2207.14780 (2022).
[B] Yang, Xinge, et al. "End-to-end hybrid refractive-diffractive lens design with differentiable ray-wave model." SIGGRAPH Asia 2024 Conference Papers. 2024.
[C] Sitzmann, Vincent, et al. "End-to-end optimization of optics and image processing for achromatic extended depth of field and super-resolution imaging." ACM Transactions on Graphics (TOG) 37.4 (2018): 1-13.
[D] Liu, Renhao, et al. "Recovery of continuous 3D refractive index maps from discrete intensity-only measurements using neural fields." Nature Machine Intelligence 4.9 (2022): 781-791.

The manuscript states "INRs promise to improve reconstruction quality on smaller amounts of data, as they can be pre-trained on large datasets". The vast majority of INRs methods are fully self-supervised and are NOT pretrained on large datasets. With a few exceptions [E,F], INRs cannot be pretrained. The strength of INRs cannot be attributed to pretraining.
[E] Dupont, Emilien, et al. "From data to functa: Your data point is a function and you can treat it like one." International Conference on Machine Learning. PMLR, 2022.
[F] Vyas, Kushal Kardam, et al. "Learning transferable features for implicit neural representations." Advances in Neural Information Processing Systems 37 (2024): 42268-42291.

The manuscript states "Classically, inverse problems such as phase retreival rely on solving an approximate linearized system". It's inaccurate to claim alternating projection methods like Gerchberg-Saxon are linearizing the phase retrieval problem. Most phase retrieval algorithms used today do not rely on linearization.

The discussion around porting wave simulation code to GPUs (line 177) makes the problem sound harder than it is. This section presents Matlab and numpy as the SOTA, while the more appropriate competitors are pytorch and tensorflow, which are trivial to map to GPUs. This section should clarify what Chromatix and JAX offer over implementations in those languages.

The statement "Chromatix also supports implicit neural representations (INRs) that in some cases improve the optimization loss landscape" needs evidence. Presumably a citation to [39].

It would be useful to explicitly discuss the limitations of the method.

Reviewer #1 (Remarks on code availability):

Code is well documented and offers many capabilities

Reviewer #2 (Remarks to the Author):

Deb, Both, and Turaga et al introduced a powerful wave optics simulation and optimization tool utilizing a recently developed parallel computation toolbox, JAX. This combination resulted in a novel and exciting toolkit for all optical system developers and especially those working on microscopy. Chromatix stands quite unique in terms of its focus compared to other existing packages, some of which are decades old, in terms of its wave optics focus, integrated parallelization, and differentiation, and thus the potential to unify computational efforts of wave optics simulations from individual labs into a single platform.

The reviewer has tested the Chromatix package according to their online documentation. It is designed quite elegantly and different categories, including fields, elements, microscope, etc., facilitate understanding during coding and development. I believe this work will gather much attention from microscopy experts and also provide broad access to labs where such a tool is missing. I recommend publication of this work in Nature Methods after the authors address the following comments.

1. Some of the function inputs are complex and unintuitive. It usually is stated with abbreviations such as [1 B ... H W], etc, in the online documentation without further explanation. The only way to know how to use these functions is from their provided example or by looking at the source code. It is crucial to provide sufficient explanation on inputs and outputs with format requirements/units. Without it, the uses of the researchers will be limited to the examples provided by the authors.

2. The designed 4f system requires two lenses to have equal focal lengths. This typically isn't implemented, but rather f1=/=f2 in microscopy systems where magnification needs to be adjusted. It would be useful if the authors could make this function for flexible.

Reviewer #2 (Remarks on code availability):

My comment on the code is included in the above comments.

Version 1:

Decision Letter:

Our ref: NMETH-A61210A

9th Jan 2026

Dear Srini,

Thank you for submitting your revised manuscript "Chromatix: a differentiable, GPU-accelerated wave-optics library" (NMETH-A61210A). It has now been seen by the original referees and their comments are below. The reviewers find that the paper has improved in revision, and therefore we'll be happy in principle to publish it in Nature Methods, pending minor revisions to comply with our editorial and formatting guidelines.

We are now performing detailed checks on your paper and will send you a checklist detailing our editorial and formatting requirements within two weeks or so. Please do not upload the final materials and make any revisions until you receive this additional information from us.

TRANSPARENT PEER REVIEW
Nature Methods offers a transparent peer review option for new original research manuscripts. We encourage increased transparency in peer review by publishing the reviewer comments, author rebuttal letters and editorial decision letters if the authors agree. Such peer review material is made available as a supplementary peer review file. **Please state in the cover letter 'I wish to participate in transparent peer review' if you want to opt in, or 'I do not wish to participate in transparent peer review' if you don't.** Failure to state your preference will result in delays in accepting your manuscript for publication.

Please note: we allow redactions to authors' rebuttal and reviewer comments in the interest of confidentiality. If you are concerned about the release of confidential data, please let us know specifically what information you would like to have removed. Please note that we cannot incorporate redactions for any other reasons. Reviewer names will be published in the peer review files if the reviewer signed the comments to authors, or if reviewers explicitly agree to release their name. For more information, please refer to our FAQ page.

ORCID
IMPORTANT: Non-corresponding authors do not have to link their ORCIDs but are encouraged to do so. Please note that it will not be possible to add/modify ORCIDs at proof. Thus, please let your co-authors know that if they wish to have their ORCID added to the paper they must follow the procedure described in the following link prior to acceptance: https://www.springernature.com/gp/researchers/orcid/orcid-for-nature-research

Author names using non-Roman characters
Nature Portfolio journals can support presentation of author names using non-Roman characters in the HTML version of the article. If you wish to, please include author names in parentheses after the Roman-character spelling; see example online here. Currently supported scripts are: Arabic, Chinese, Cyrillic, Devanagari, Greek, Hebrew, Hangul, Japanese and Persian. You will be asked to verify the rendering is correct at proof stage.

Thank you again for your interest in Nature Methods. Please do not hesitate to contact me if you have any questions. We will be in touch again soon.

Best regards,
Nina

Nina Vogt, PhD
Senior Editor
Nature Methods

Reviewer #1 (Remarks to the Author):

The revision has adequately addressed all my concerns; the contributions are now much better contextualized. I support publication of the manuscript in its current form.

Reviewer #1 (Remarks on code availability):

The code is reasonably well documented. My students have been able to run it and apply it to their problems without issue.

Reviewer #2 (Remarks to the Author):

The authors have provided sufficient revisions addressing the reviewers' comments. I consider this version of the manuscript acceptable for publication.

Reviewer #2 (Remarks on code availability):

Code and instructions facilitate usage and provided demo on the software website is useful to initialize projects.

Open Access This Peer Review File is licensed under a Creative Commons Attribution 4.0 International License, which permits use, sharing, adaptation, distribution and reproduction in any medium or format, as long as you give appropriate credit to the original author(s) and the source, provide a link to the Creative Commons license, and indicate if changes were made.
In cases where reviewers are anonymous, credit should be given to 'Anonymous Referee' and the source.
The images or other third party material in this Peer Review File are included in the article's Creative Commons license, unless indicated otherwise in a credit line to the material. If material is not included in the article's Creative Commons license and your intended use is not permitted by statutory regulation or exceeds the permitted use, you will need to obtain permission directly from the copyright holder.
To view a copy of this license, visit https://creativecommons.org/licenses/by/4.0/

We thank both reviewers for their comments and respond to each of their points below:

Reviewer #1:

This manuscript introduces Chromatix, an open-source, JAX-based, GPU-accelerated, and differentiable wave-optics simulation library. While many differentiable wave-optics simulators exist, Chromatix generally offers a more complete feature set and, largely (entirely?) due to its JAX-based implementation, is more efficient and parallelizable than existing methods. Chromatix is benchmarked on a range of computational microscopy problems and is shown to offer consistent acceleration of >2x on one GPU and up to 22x on many GPUs. Its parallelism also allows it to tackle larger, higher-resolution problems than existing methods can handle. Chromatix is validated on an impressive number of computational microscopy problems including spatially-varying deconvolution, phase retrieval, PSF engineering, and computer generated holography.

While not groundbreaking (similar though less performant software has existed for some time), Chromatix could still be of value to the computational imaging community. The support for multi-GPU acceleration is a particularly valuable contribution; it enables higher-resolution reconstructions than are possible with alternative methods.

Comments:
The discussion of related work is lacking. It overlooks several recent works that offer capabilities that are not supported by Chromatix. For instance, [A] supports metaoptics design and [B] supports differentiable hybrid ray-wave based methods are fast enough to enable the design of compound optics. The manuscript also overlooks [C] and a large body of follow-up works that used differentiable end-to-end optical models to design computational imaging systems. Other early works that used differentiable optical models for computational microscopy problems are also overlooked [D]. The discussion of CGH is also somewhat sparse.

[A] D-flat: Hazineh, Dean S., et al. "D-flat: A differentiable flat-optics framework for end-to-end metasurface visual sensor design." arXiv preprint arXiv:2207.14780 (2022).
[B] Yang, Xinge, et al. "End-to-end hybrid refractive-diffractive lens design with differentiable ray-wave model." SIGGRAPH Asia 2024 Conference Papers. 2024.
[C] Sitzmann, Vincent, et al. "End-to-end optimization of optics and image processing for achromatic extended depth of field and super-resolution imaging." ACM Transactions on Graphics (TOG) 37.4 (2018): 1-13.
[D] Liu, Renhao, et al. "Recovery of continuous 3D refractive index maps from discrete intensity-only measurements using neural fields." Nature Machine Intelligence 4.9 (2022): 781-791.

We thank the reviewer for these suggestions. References [C] and [D] have been added to the text along with discussion of other related work implementing end-to-end deep learning

optimization of optical models (lines 72 - 76). We have included more background on the use of neural networks for holography when introducing DeepCGH (lines 376 - 387). We have also added references to [A] and [B] and want to highlight these:

[A]: D-flat is a differentiable method specifically for optimizing metasurfaces. So far Chromatix has focused on biological applications, and none of our users utilize metasurfaces. We think this could be a great new area for Chromatix and we are grateful for this reference. We have recently added early support for the Modified Born Series, which is able to model meta-optics without approximations, although its convergence behaviour is less than ideal for metasurfaces. The provided reference bridges that gap, and we have added it to our discussion on limitations and outlook (lines 523 - 526).

[B]: This paper shows how to combine ray tracing models with diffractive modelling. Indeed a big limitation of Chromatix is its (current) limitation to diffractive effects, and this paper would allow us to integrate ray tracing models. We think this is a very exciting direction in making Chromatix more general and would like to start exploring this soon; we have added it to our discussion on limitations and outlook (lines 519 - 521).

> *The manuscript states "INRs promise to improve reconstruction quality on smaller amounts of data, as they can be pre-trained on large datasets". The vast majority of INRs methods are fully self-supervised and are NOT pretrained on large datasets. With a few exceptions [E,F], INRs cannot be pretrained. The strength of INRs cannot be attributed to pretraining.*
> *[E] Dupont, Emilien, et al. "From data to functa: Your data point is a function and you can treat it like one." International Conference on Machine Learning. PMLR, 2022.*
> *[F] Vyas, Kushal Kardam, et al. "Learning transferable features for implicit neural representations." Advances in Neural Information Processing Systems 37 (2024): 42268-42291.*

We agree with the reviewer that this statement needs to be removed and have updated the discussion of INRs correspondingly along with references to some of the uses prior work has found for INRs in computational optics (lines 163 - 169).

> *The manuscript states "Classically, inverse problems such as phase retreival rely on solving an approximate linearized system". It's inaccurate to claim alternating projection methods like Gerchberg-Saxon are linearizing the phase retrieval problem. Most phase retrieval algorithms used today do not rely on linearization.*

We agree that “linear” is inaccurate and have removed it (lines 153 - 155).

> *The discussion around porting wave simulation code to GPUs (line 177) makes the problem sound harder than it is. This section presents Matlab and numpy as the SOTA, while the more appropriate competitors are pytorch and tensorflow, which are trivial to*

map to GPUs. This section should clarify what Chromatix and JAX offer over implementations in those languages.

We agree that this section should be clarified, and we have included a discussion of PyTorch/TensorFlow in comparison to JAX for optical simulations here (lines 190 - 195). We have also listed these frameworks in our supplementary feature comparison table (Table S1).

The statement "Chromatix also supports implicit neural representations (INRs) that in some cases improve the optimization loss landscape" needs evidence. Presumably a citation to [39].

Indeed, we have added the citation (line 289).

It would be useful to explicitly discuss the limitations of the method.

Reviewer #1 (Remarks on code availability):
Code is well documented and offers many capabilities

We have added more discussion of the current limitations of Chromatix along with our outlook on future work to potentially address these limitations (lines 513 - 529).

Reviewer #2:

Deb, Both, and Turaga et al introduced a powerful wave optics simulation and optimization tool utilizing a recently developed parallel computation toolbox, JAX. This combination resulted in a novel and exciting toolkit for all optical system developers and especially those working on microscopy. Chromatix stands quite unique in terms of its focus compared to other existing packages, some of which are decades old, in terms of its wave optics focus, integrated parallelization, and differentiation, and thus the potential to unify computational efforts of wave optics simulations from individual labs into a single platform.

The reviewer has tested the Chromatix package according to their online documentation. It is designed quite elegantly and different categories, including fields, elements, microscope, etc., facilitate understanding during coding and development.

I believe this work will gather much attention from microscopy experts and also provide broad access to labs where such a tool is missing. I recommend publication of this work in Nature Methods after the authors address the following comments.

We're excited to see the reviewer highlight the point of broad access, as it's exactly what we designed Chromatix for!

1. Some of the function inputs are complex and unintuitive. It usually is stated with abbreviations such as `[1 B ... H W]`, etc, in the online documentation without

> *further explanation. The only way to know how to use these functions is from their provided example or by looking at the source code. It is crucial to provide sufficient explanation on inputs and outputs with format requirements/units. Without it, the uses of the researchers will be limited to the examples provided by the authors.*

We agree that the input shapes are confusing; we've also seen participants in our hackathons struggling with this. To remedy this we have upgraded the `Field` with a new design which removes all empty axes when they are not needed, making inputs and their shapes a lot simpler. We have also clarified the documentation with typed shape annotation of all the functions and explicit labels such as `(height, width)` instead of abbreviations such as `(H, W)`. This is now addressed in pull request #175 (https://github.com/chromatix-team/chromatix/pull/175) and is now the default in Chromatix.

> *2. The designed 4f system requires two lenses to have equal focal lengths. This typically isn't implemented, but rather f1=/=f2 in microscopy systems where magnification needs to be adjusted. It would be useful if the authors could make this function for flexible.*
>
> *Reviewer #2 (Remarks on code availability):*
> *My comment on the code is included in the above comments.*

We agree that this is a useful feature, and Chromatix indeed already supports this (e.g. arbitrary focal lengths can be used for the `objective_point_source` and `ff_lens` functions). The examples in the documentation initially did not demonstrate this for simplicity, but we have updated these examples (in the README, Chromatix 101, and the Holoscope example) with more realistic choices for objective and tube lens focal lengths in typical 4f systems.

Minor Changes:
We have also made very minor changes to some of the text in the manuscript, for example to improve transitions between concepts or clarify axes on plots. Additionally we have fixed the spectral imaging model in Figure 4c, e-f which previously incorrectly sampled the phase mask at different wavelengths (the result is slightly different but fundamentally unchanged). We list these changes here:

1. Grammar correction on line 203 (use "couple of lines" instead of "couple lines")
2. Be more general about ray-based optics with deep learning on line 77 (switch from "wave optics simulation" to "optics simulation" in order to match our expanded background on end-to-end methods in computational optics
3. Clarify our focus on wave optics on line 108
4. Clarify that Chromatix's support for INRs is in "addition to" rather than "instead of" voxel grids on line 288
5. Corrected interpolation of phase mask for the spectral PSF engineering in Fig. 4c, e-f
6. Clarify that holography can be used for optogenetics on line 436
7. Clarify that "vertical" axis shows relative speedup rather than "left" axis in Fig. 5 caption
